# Supplementary material for: Effect of leaf position and days post-infiltration on transient expression of colorectal cancer vaccine candidate proteins GA733-Fc and GA733-FcK in Nicotiana benthamiana plant
Source: PeerJ. 2021 Apr 7;9:e10851. doi: 10.7717/peerj.10851 (PMC8035899; doi:10.7717/peerj.10851)

**Figure S1: Glycan analysis of GA733-Fc^P^ (A) and GA733-FcK^P^ (B) proteins by High Performance Liquid Chromatography (HPLC)**

N-glycan released from GA733-Fc^P^ (A) and GA733-FcK^P^ (B) structure profiles were analyzed using HPLC. The glycan structures are displayed according to each peak in the graph. GlcNAc, the square; mannose, the white circle; α(1,3)-fucose, double diamond; β(1,2)-xylose, the white triangle.


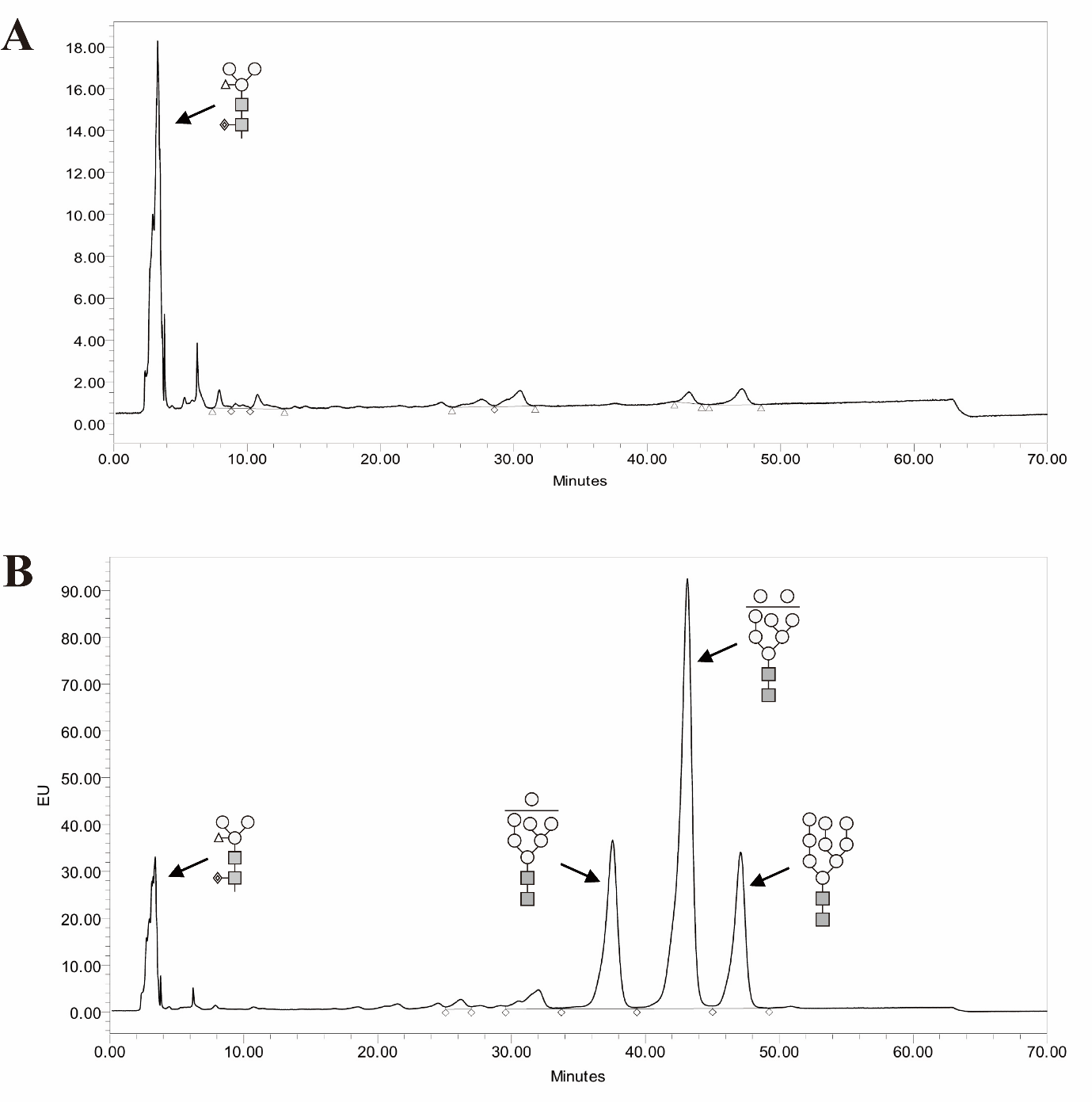


**Figure S2: Surface plasmon resonance (SPR) analysis to confirm the binding affinity of GA733-Fc^P^ (a) and GA733-FcK^P^ (b) to FcγRⅠ (CD64)**

Each curve shows concentrations of 1.5625, 3.125, 6.25, 12.5, 25, 50, and 100 nM of GA733-Fc^P^ (**A**), and GA733-FcK^P^ (**B**). His-tag antibody and FcγRⅠ (CD64) were immobilized on the chip, and GA733-Fc and GA733-FcK were used as analytes. As the concentration of analytes (GA733-Fc and GA733-FcK) decreased, the graph changed constantly. GA733-Fc^M^ did not bind. The association and dissociation rates of GA733-FcK (**B**) were higher than those of GA733-Fc (**A**). Kinetic analysis of binding affinity of GA733-Fc and GA733-FcK purified from plants to recombinant human FcγRⅠ/CD64 was conducted using SPR analysis. The peak of GA733-FcK decreased less than that of GA733-Fc. These results show that the association rate constant, dissociation rate constant, and equilibrium dissociation rate (ratio of dissociation rate constant and association rate constant from kinetic experiments) of GA733-FcK were higher than those of GA733-Fc.


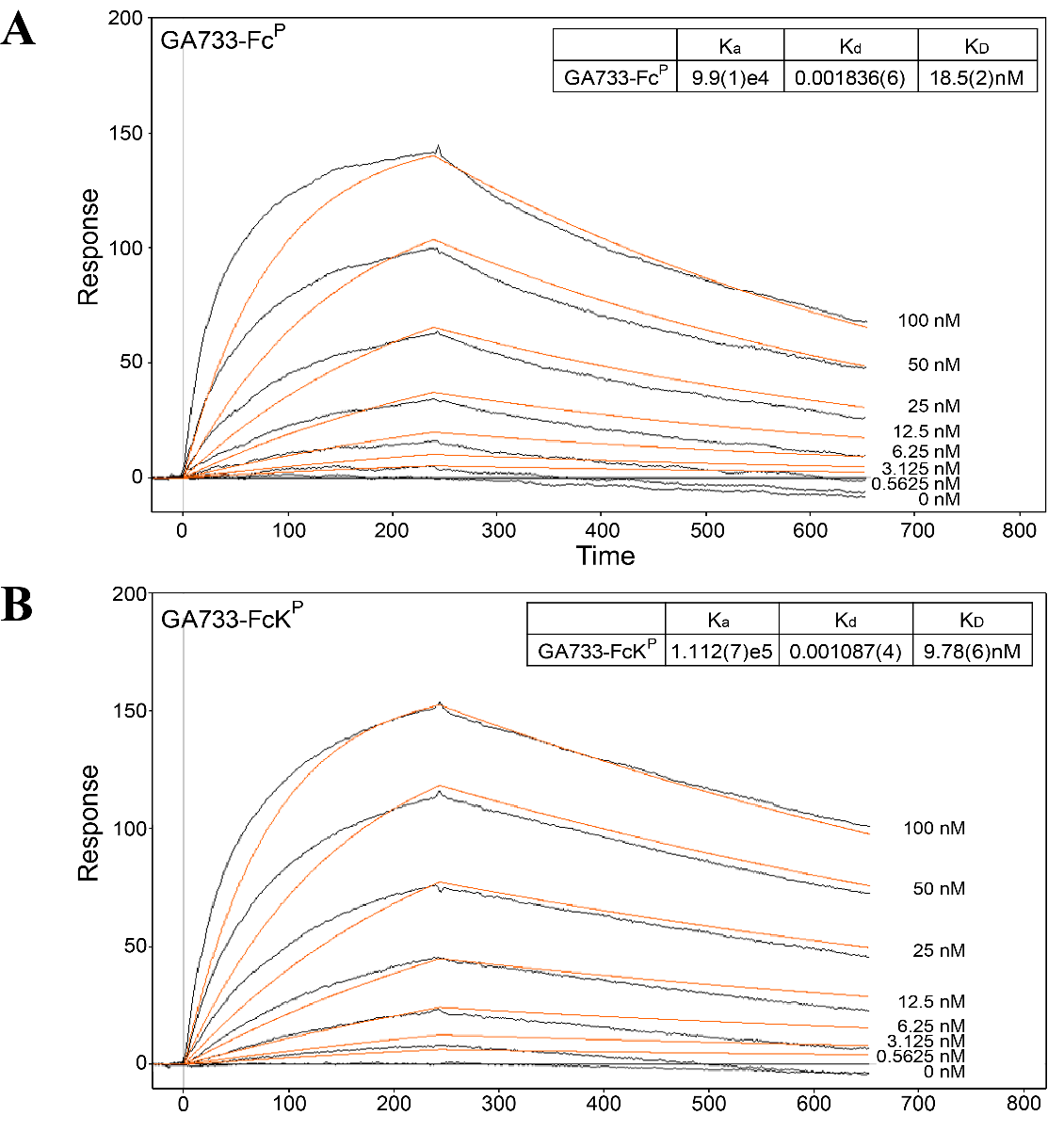

Supplement: Supplemental Information 1 [file peerj-09-10851-s001.docx]
