# Supplementary figures and images for: Effect of leaf position and days post-infiltration on transient expression of colorectal cancer vaccine candidate proteins GA733-Fc and GA733-FcK in Nicotiana benthamiana plant
Source: PeerJ. 2021 Apr 7;9:e10851. doi: 10.7717/peerj.10851 (PMC8035899; doi:10.7717/peerj.10851)

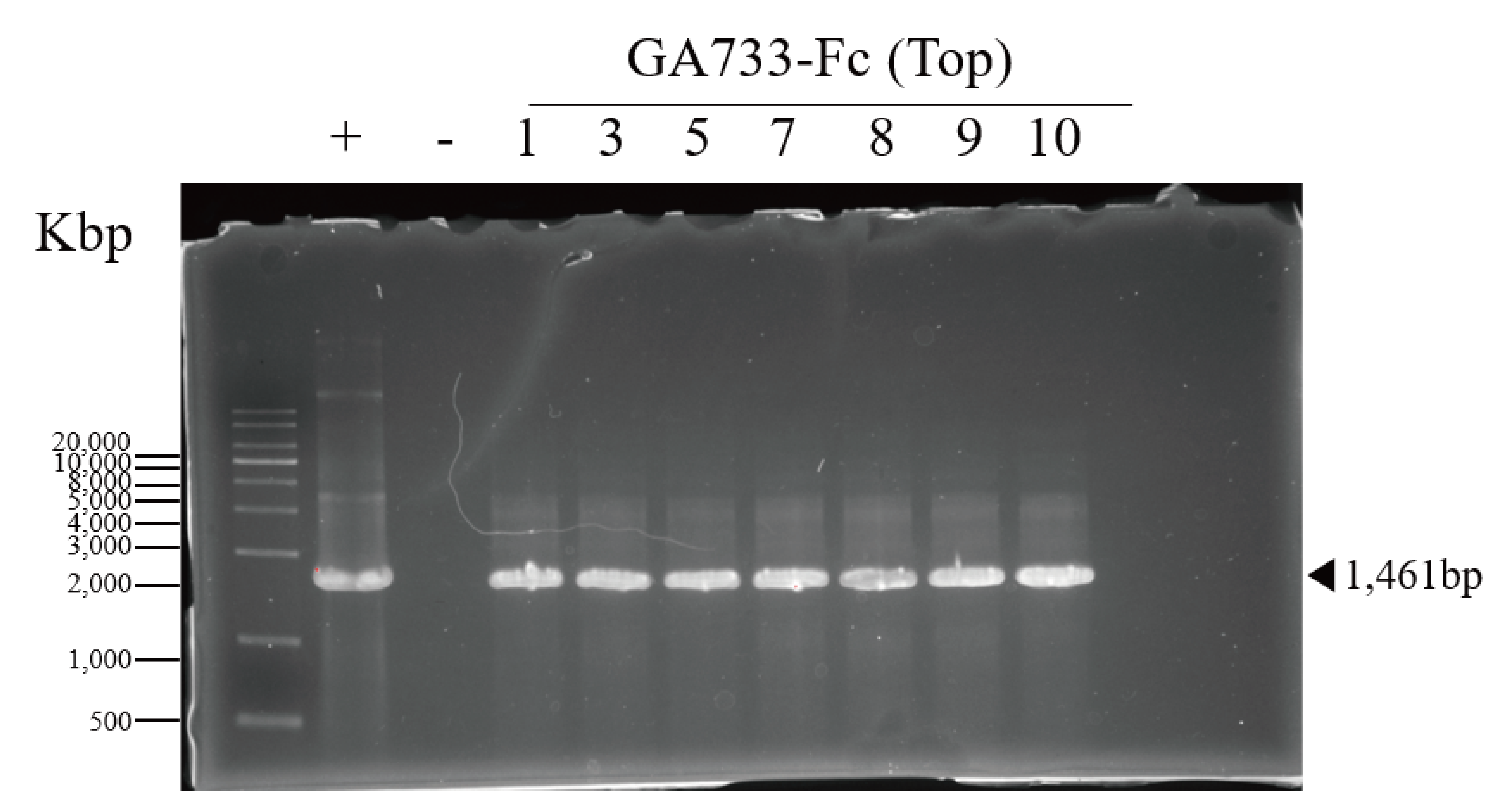

Supplement: Supplemental Information 2 — The genomic DNA fragments were extracted from infiltrated leaves located base position, amplified and separated on a 1% agarose gel using electrophoresis. Positive control (+), GA733-Fc plasmid DNA extracted from Escherichia coli negative control (-), genomic DNA extracted from non-infiltrated Nicotiana benthamiana plant top leaves; 1-10 dpi, genomic DNA extracted from infiltrated Agrobacterial inoculum carrying pEAQ-GA733-Fc (Ag/pEAQ-GA733-Fc). [file peerj-09-10851-s002.png]

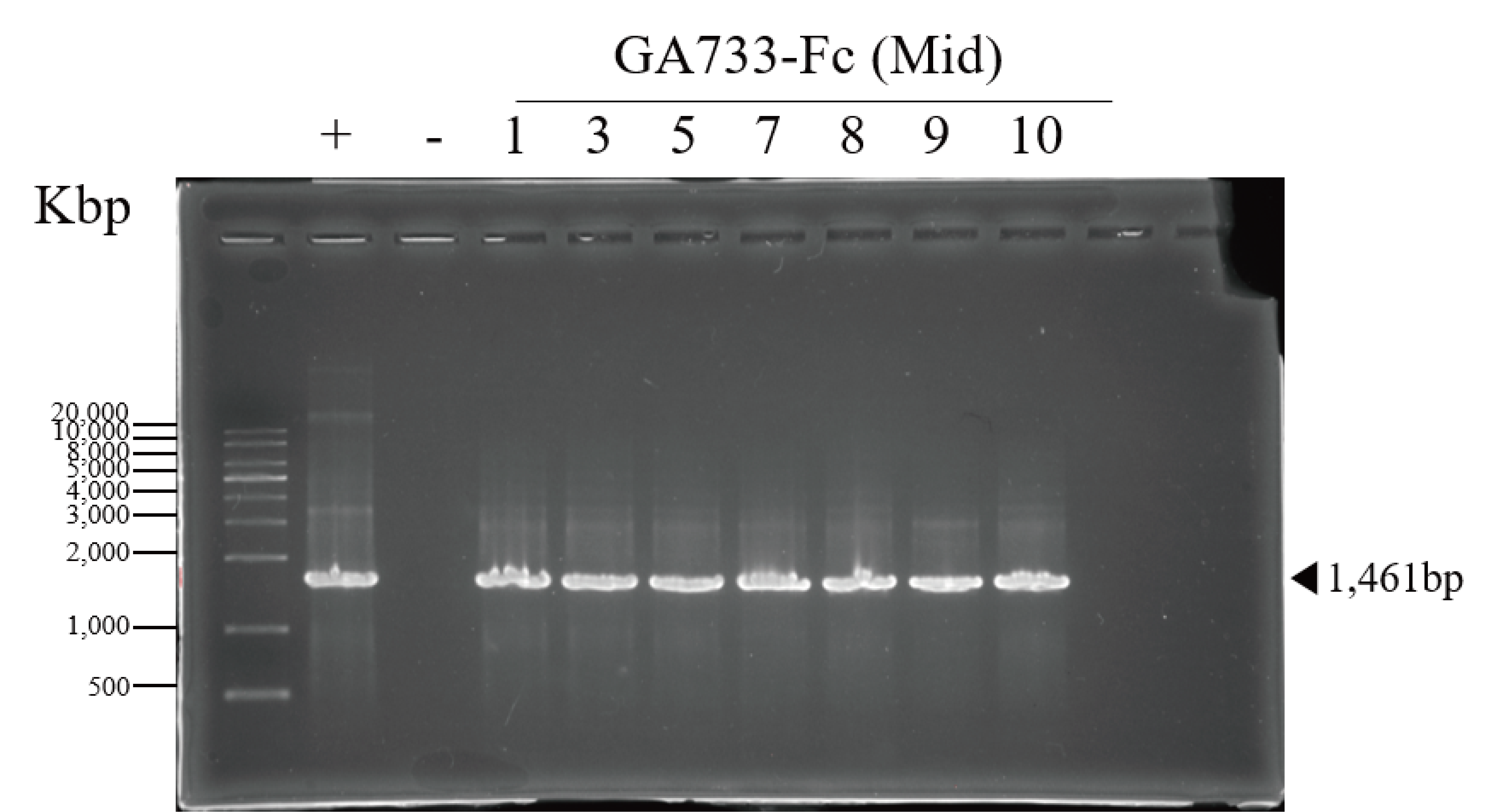

Supplement: Supplemental Information 3 — The genomic DNA fragments were extracted from infiltrated leaves located base position, amplified and separated on a 1% agarose gel using electrophoresis. Positive control (+), GA733-Fc plasmid DNA extracted from Escherichia coli; negative control (-), genomic DNA extracted from non-infiltrated Nicotiana benthamiana plant mid leaves; 1-10 dpi, genomic DNA extracted from infiltrated Agrobacterial inoculum carrying pEAQ-GA733-Fc (Ag/pEAQ-GA733-Fc). [file peerj-09-10851-s003.png]

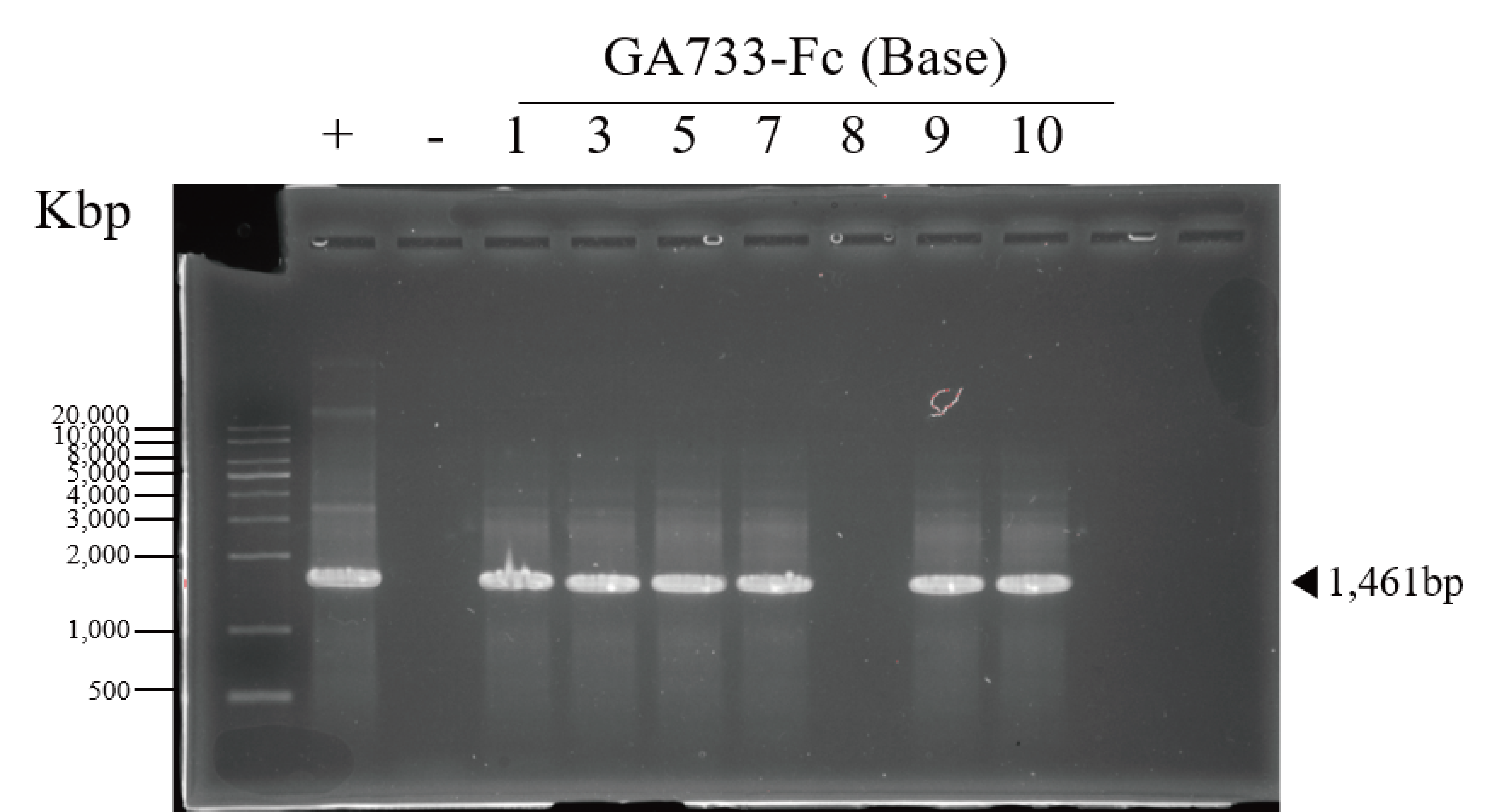

Supplement: Supplemental Information 4 — The genomic DNA fragments were extracted from infiltrated leaves located base position, amplified and separated on a 1% agarose gel using electrophoresis. Positive control (+), GA733-Fc plasmid DNA extracted from Escherichia coli; negative control (-), genomic DNA extracted from non-infiltrated Nicotiana benthamiana plant base leaves; 1-10 dpi, genomic DNA extracted from infiltrated Agrobacterial inoculum carrying pEAQ-GA733-Fc (Ag/pEAQ-GA733-Fc). [file peerj-09-10851-s004.png]

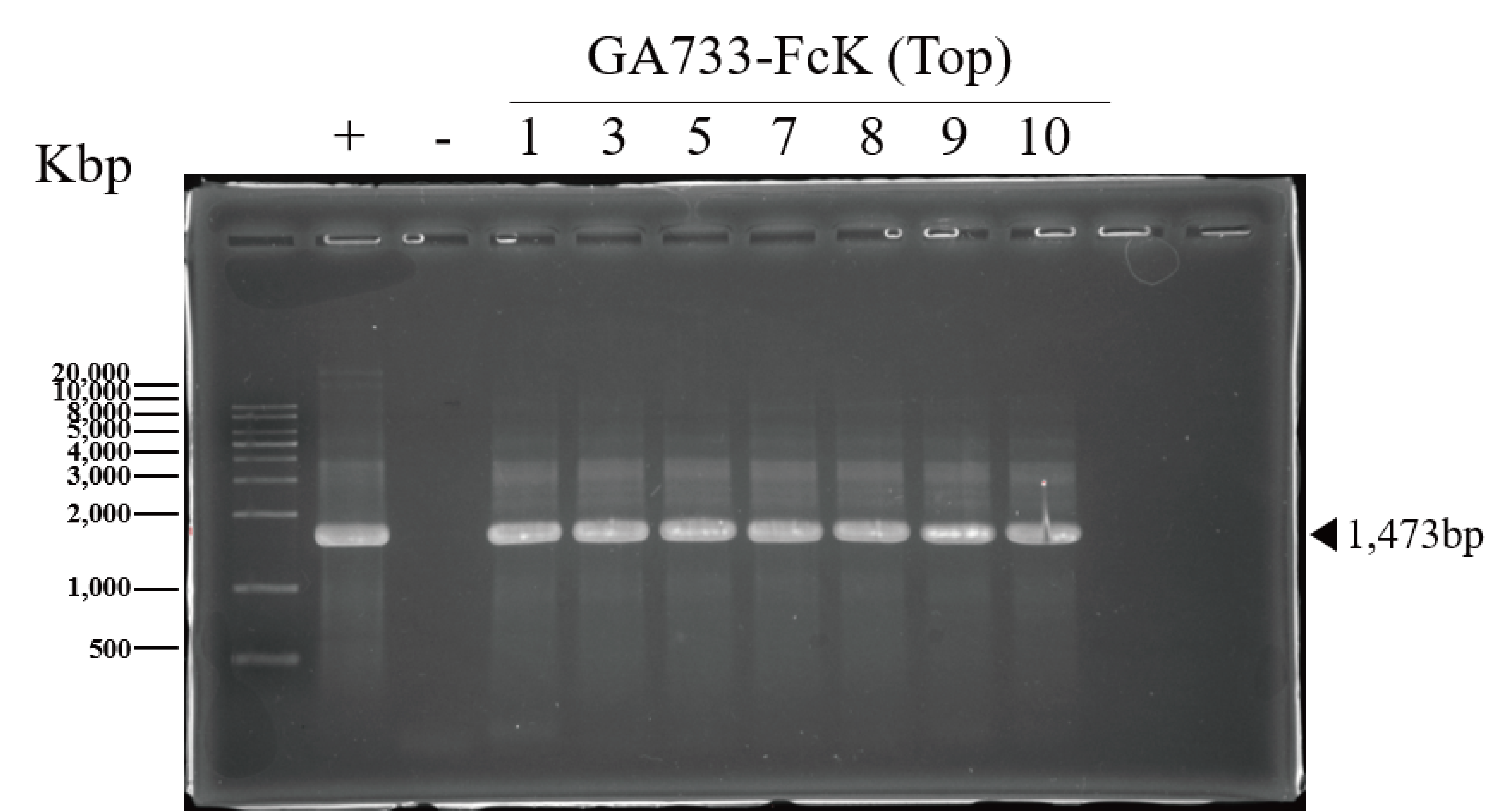

Supplement: Supplemental Information 5 — The genomic DNA fragments were extracted from infiltrated leaves located base position, amplified and separated on a 1% agarose gel using electrophoresis. Positive control (+), GA733-FcK plasmid DNA extracted from Escherichia coli; negative control (-), genomic DNA extracted from non-infiltrated Nicotiana benthamiana plant top leave; 1-10 dpi, genomic DNA extracted from infiltrated Agrobacterial inoculum carrying pEAQ-GA733-FcK (Ag/pEAQ-GA733-FcK). [file peerj-09-10851-s005.png]

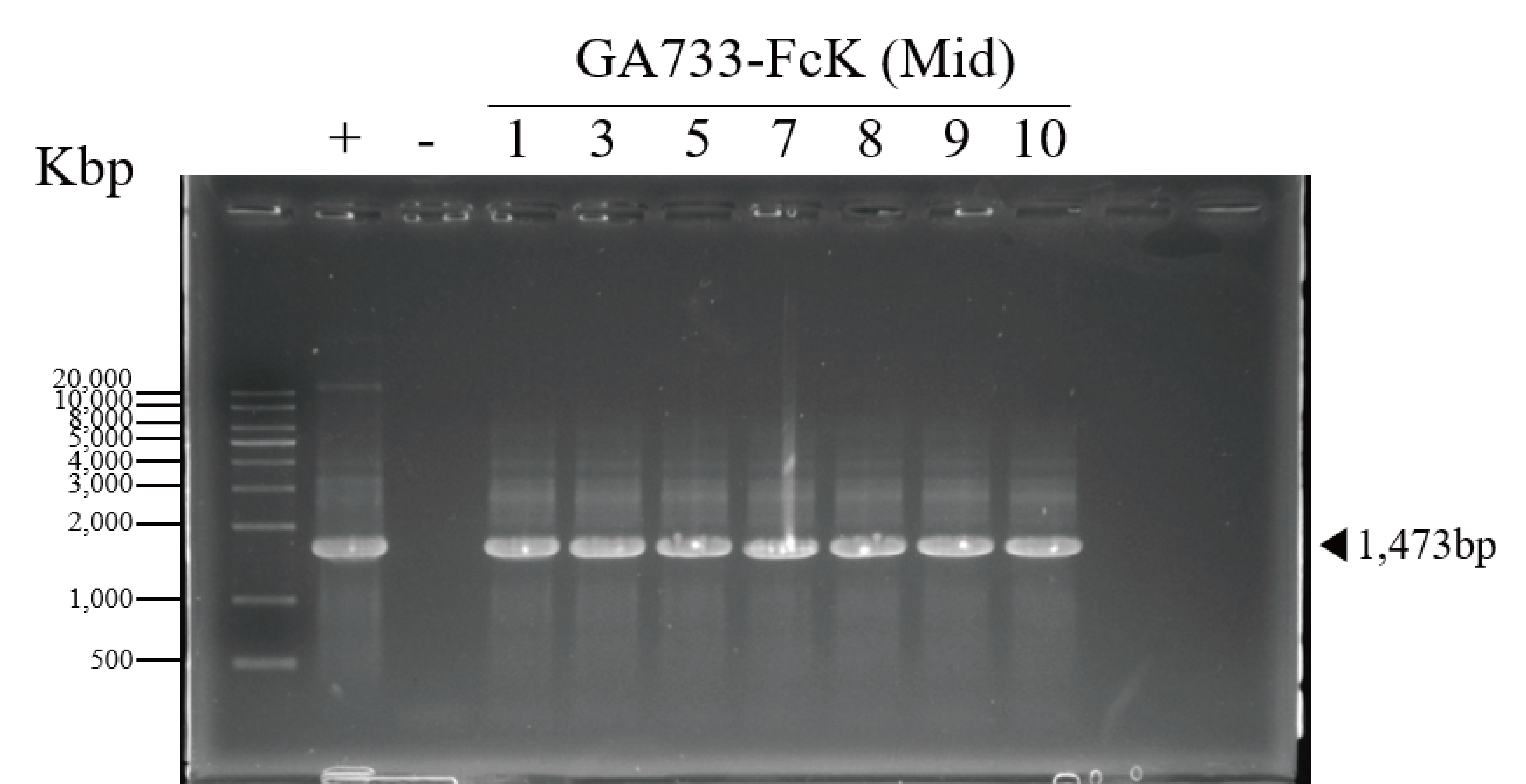

Supplement: Supplemental Information 6 — The genomic DNA fragments were extracted from infiltrated leaves located base position, amplified and separated on a 1% agarose gel using electrophoresis. Positive control (+), GA733-FcK plasmid DNA extracted from Escherichia coli; negative control (-), genomic DNA extracted from non-infiltrated Nicotiana benthamiana plant base leaves; 1-10 dpi, genomic DNA extracted from infiltrated Agrobacterial inoculum carrying pEAQ-GA733-FcK (Ag/pEAQ-GA733-FcK). [file peerj-09-10851-s006.png]

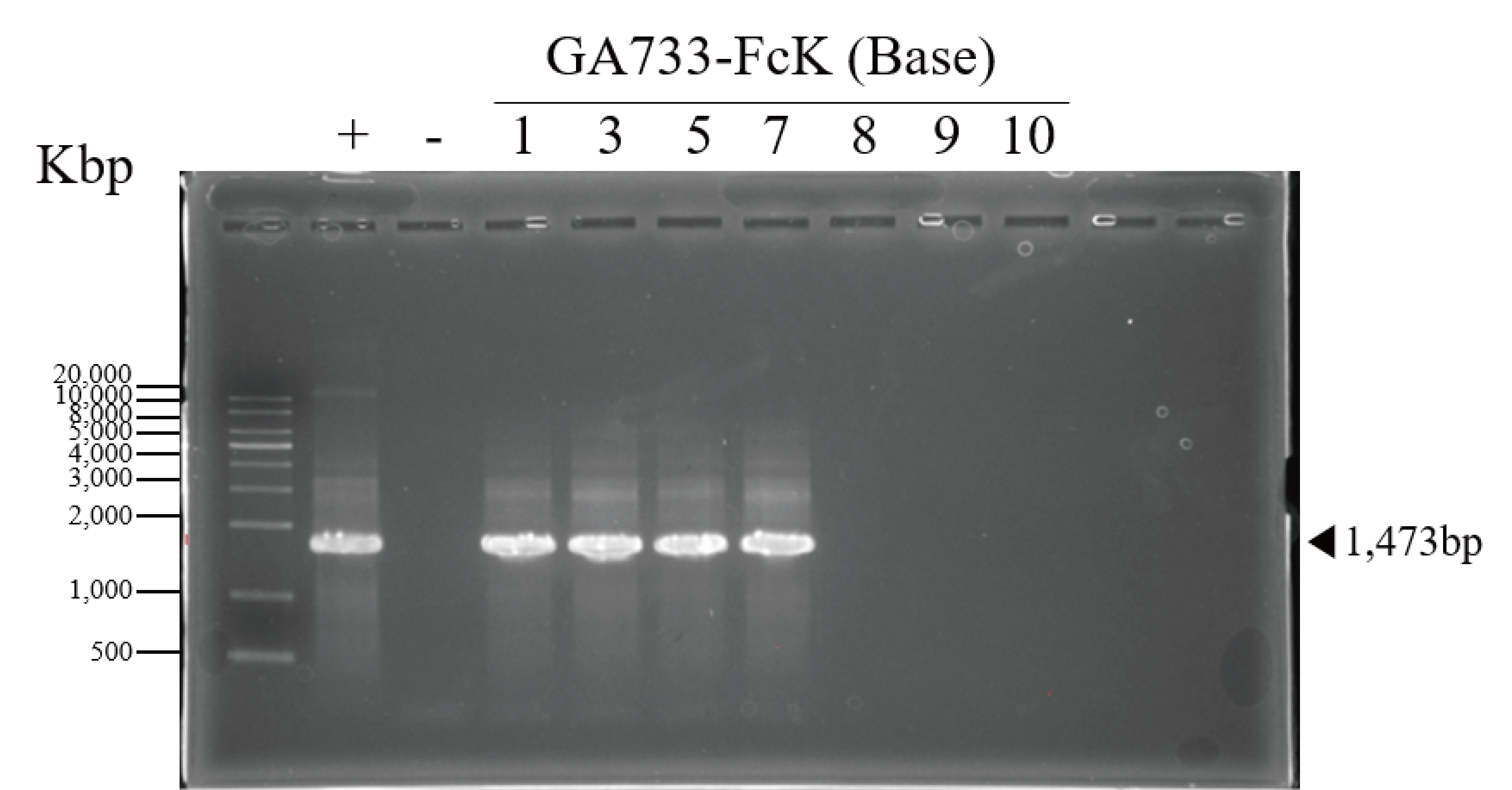

Supplement: Supplemental Information 7 — The genomic DNA fragments were extracted from infiltrated leaves located base position, amplified and separated on a 1% agarose gel using electrophoresis. Positive control (+), GA733-FcK plasmid DNA extracted from Escherichia coli; negative control (-), genomic DNA extracted from non-infiltrated Nicotiana benthamiana plant mid leaves; 1-10 dpi, genomic DNA extracted from infiltrated Agrobacterial inoculum carrying pEAQ-GA733-FcK (Ag/pEAQ-GA733-FcK). [file peerj-09-10851-s007.png]

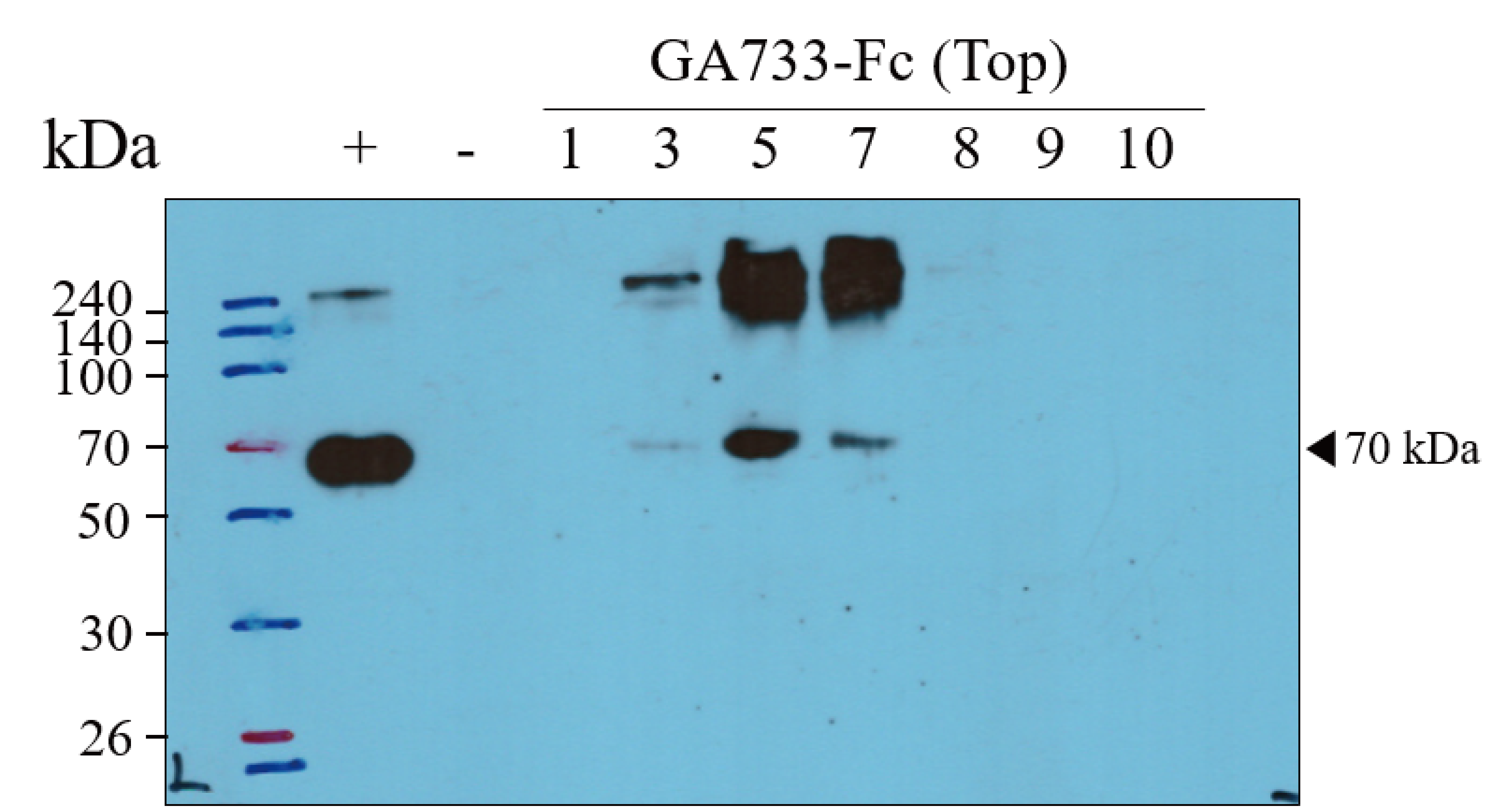

Supplement: Supplemental Information 8 — Lane 1, protein marker; Lane 2, positive control (+), mammalian-derived GA733-Fc (EpCAM-Fc M), 70 ng; -, negative control (non-infiltrated Nicotiana benthamiana plant base leaves); 1-10 dpi, samples. [file peerj-09-10851-s008.png]

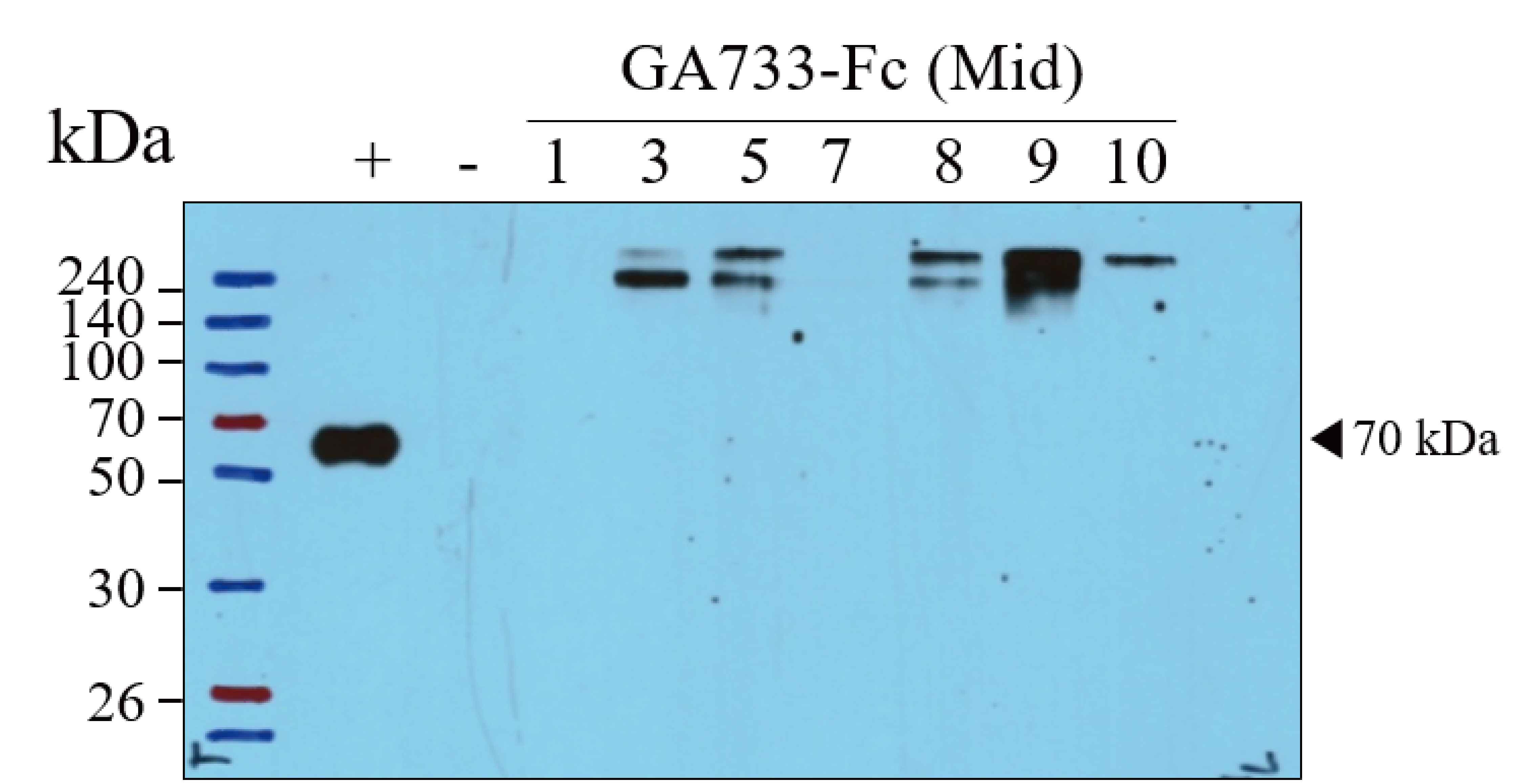

Supplement: Supplemental Information 9 — Lane 1, protein marker; Lane 2, positive control (+), mammalian-derived GA733-Fc (EpCAM-Fc M), 70 ng; -, negative control (non-infiltrated Nicotiana benthamiana plant base leaves); 1-10 dpi, samples. [file peerj-09-10851-s009.png]

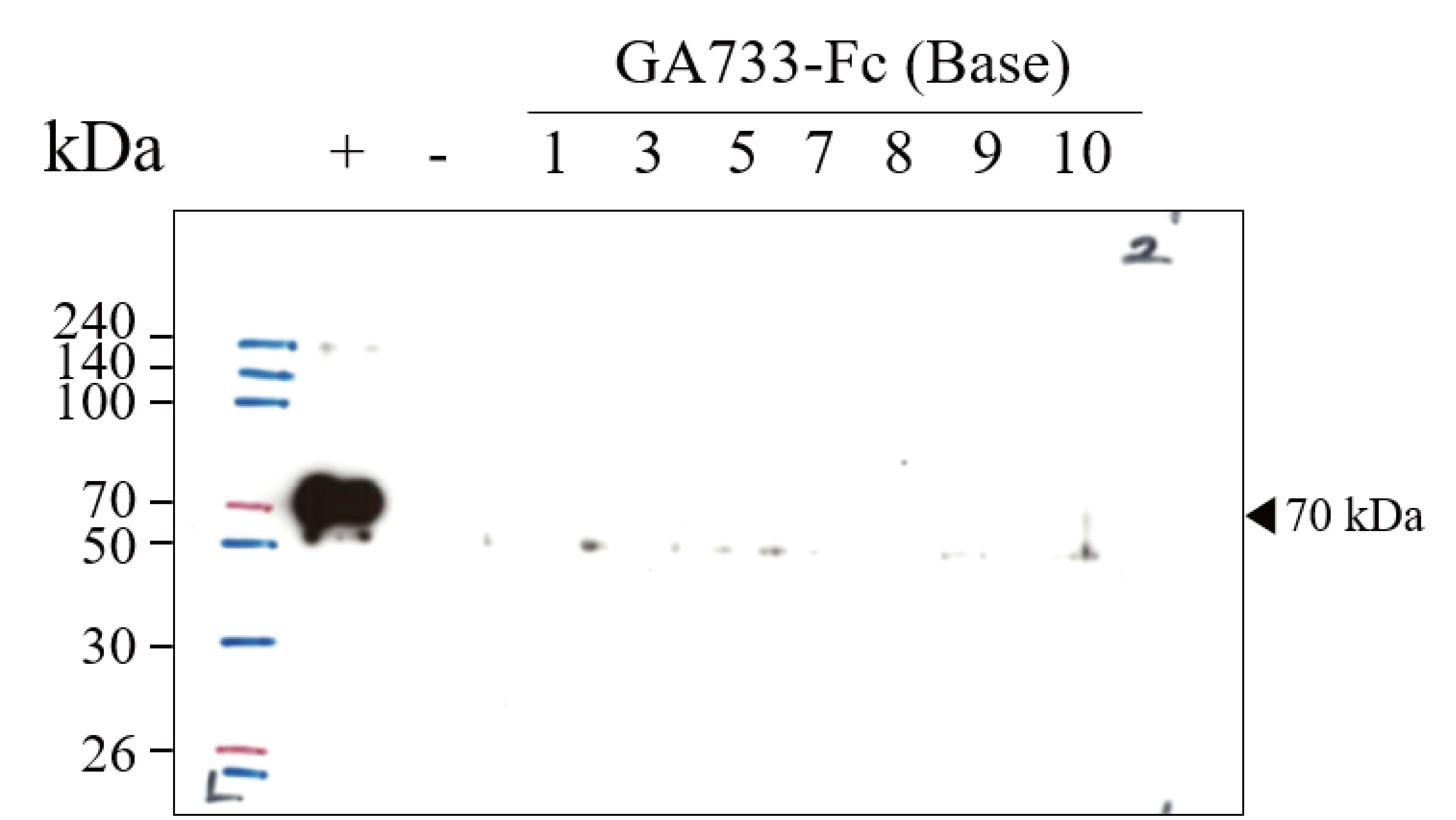

Supplement: Supplemental Information 10 — Lane 1, protein marker; Lane 2, positive control (+), mammalian-derived GA733-Fc (EpCAM-Fc M), 70 ng; -, negative control (non-infiltrated Nicotiana benthamiana plant base leaves); 1-10 dpi, samples. [file peerj-09-10851-s010.png]

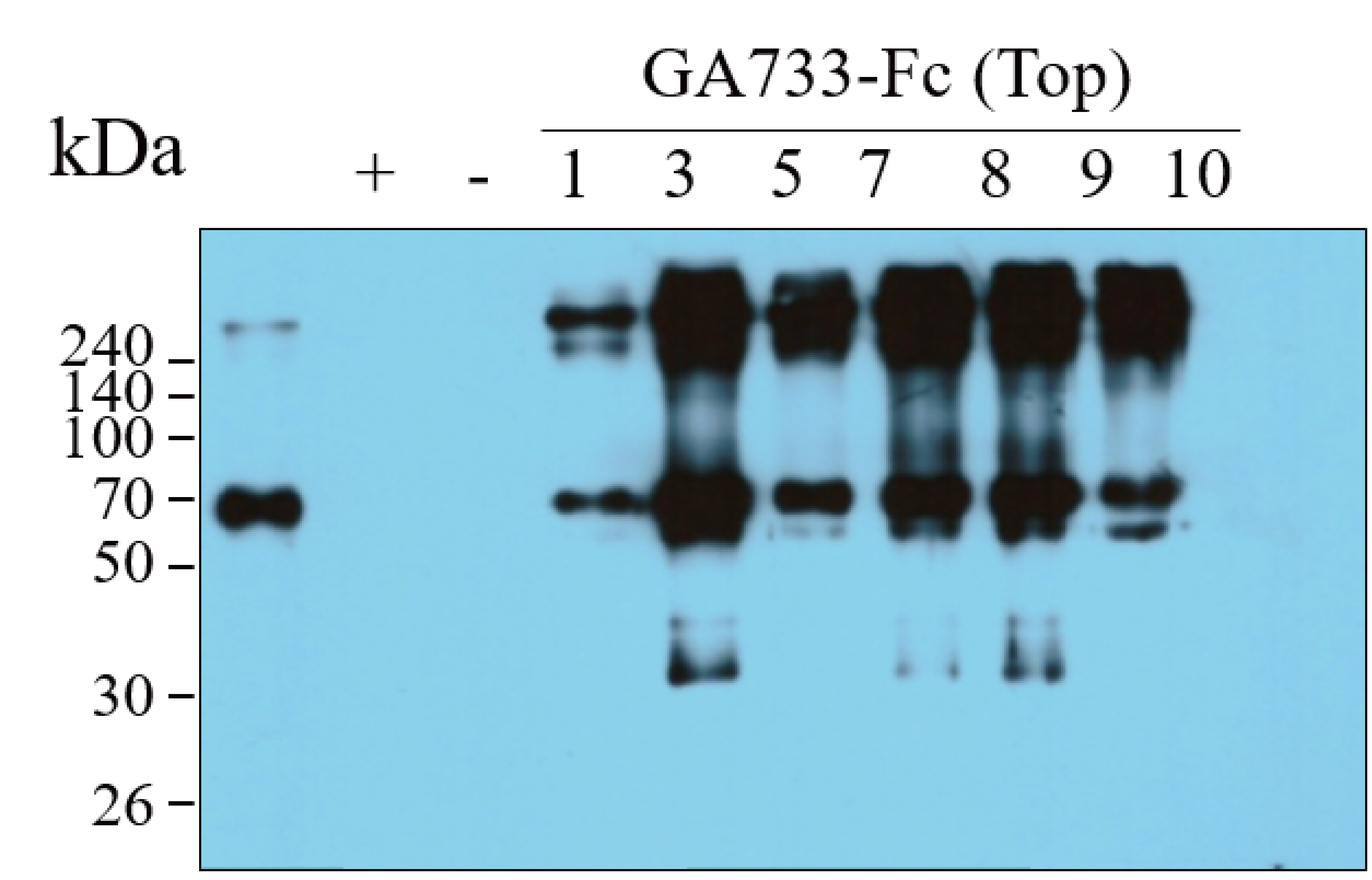

Supplement: Supplemental Information 11 — Lane 1, protein marker; Lane 2, positive control (+), mammalian-derived GA733-Fc (EpCAM-Fc M), 70 ng; -, negative control (non-infiltrated Nicotiana benthamiana plant base leaves); 1-10 dpi, samples. [file peerj-09-10851-s011.png]

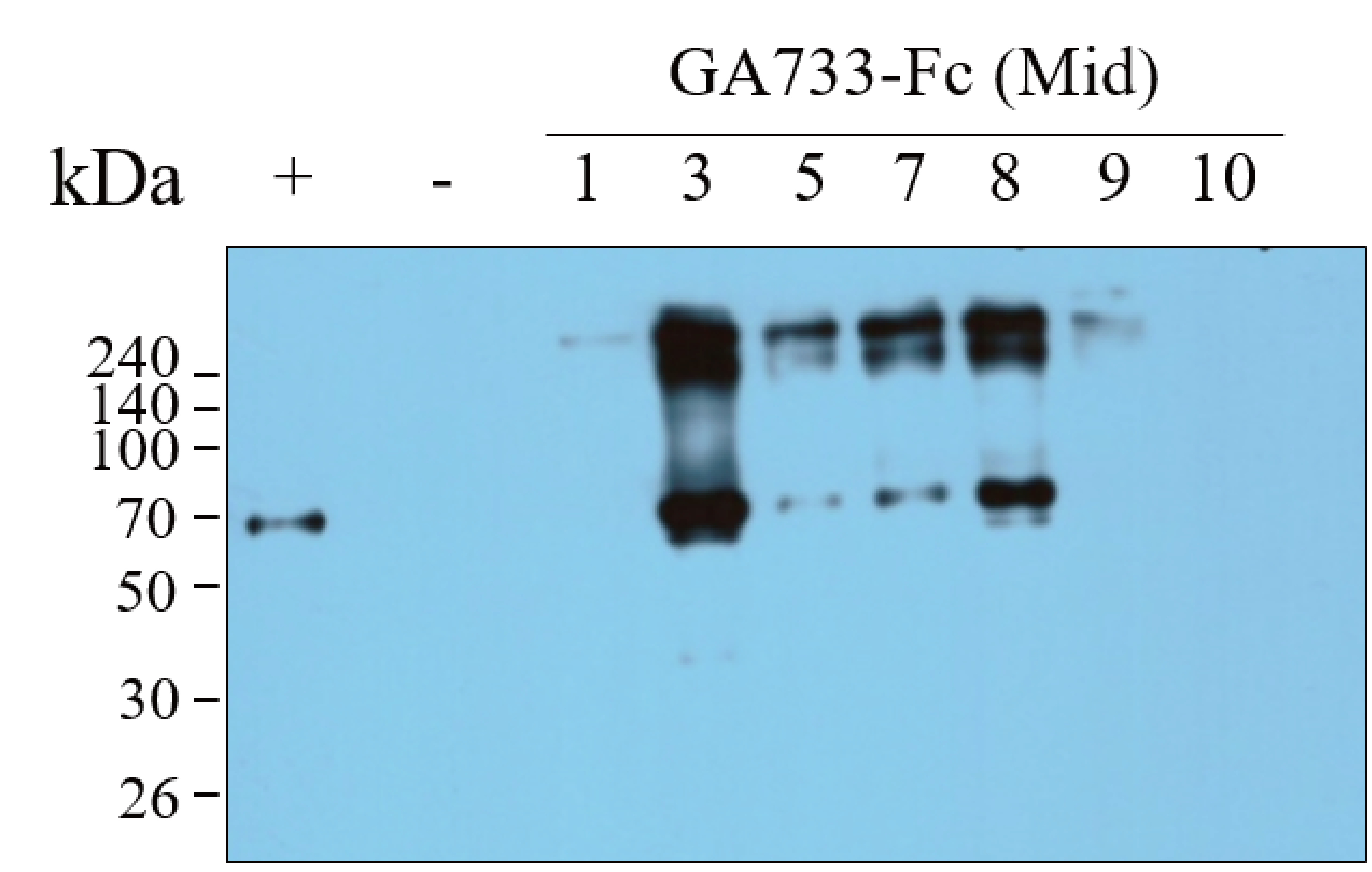

Supplement: Supplemental Information 12 — Lane 1, protein marker; Lane 2, positive control (+), mammalian-derived GA733-Fc (EpCAM-Fc M), 70 ng; -, negative control (non-infiltrated Nicotiana benthamiana plant base leaves); 1-10 dpi, samples. [file peerj-09-10851-s012.png]

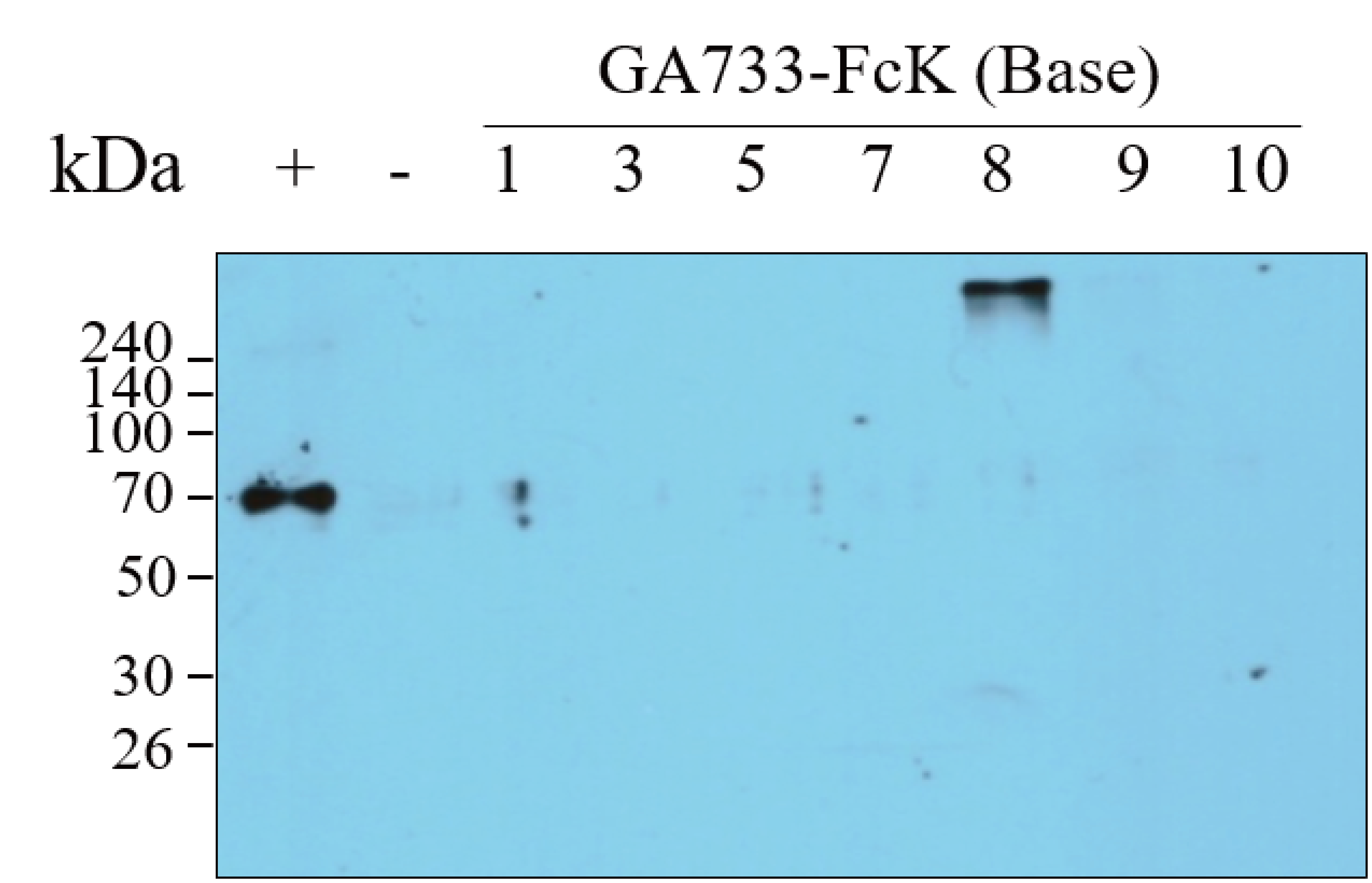

Supplement: Supplemental Information 13 — Lane 1, protein marker; Lane 2, positive control (+), mammalian-derived GA733-Fc (EpCAM-Fc M), 70 ng; -, negative control (non-infiltrated Nicotiana benthamiana plant base leave); 1-10 dpi, samples. [file peerj-09-10851-s013.png]

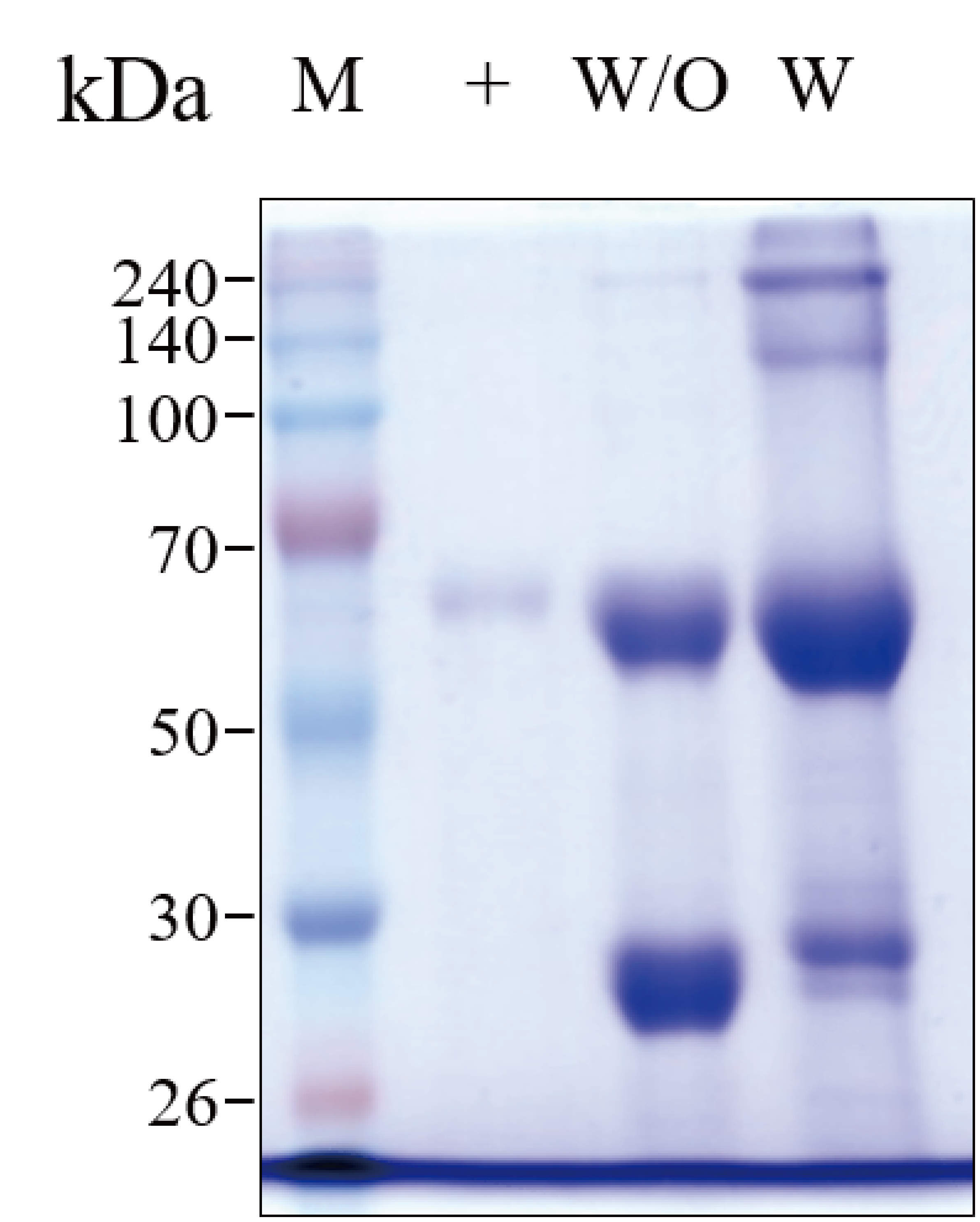

Supplement: Supplemental Information 15 — Lane 1, protein marker; Lane 2, positive control (+), mammalian-derived GA733-Fc (EpCAM-Fc M), 100 μg; Lane 3, The purified GA733-Fc P; Lane 4 The purified GA733-FcK P. Commercial protein A resin column (Sepharose 4 Fast Flow, GE Healthcare, Sweden, NJ). The gel was stained with Coomassie blue staining solution [10% acetic acid (v/v), 30% methanol (v/v), and 0.01% Coomassie blue (w/v)] at room temperature for 30 min with shaking. The gel was destained with destaining solution (10% acetic acid and 30% methanol) thrice for 30 min each withshaking. [file peerj-09-10851-s015.png]

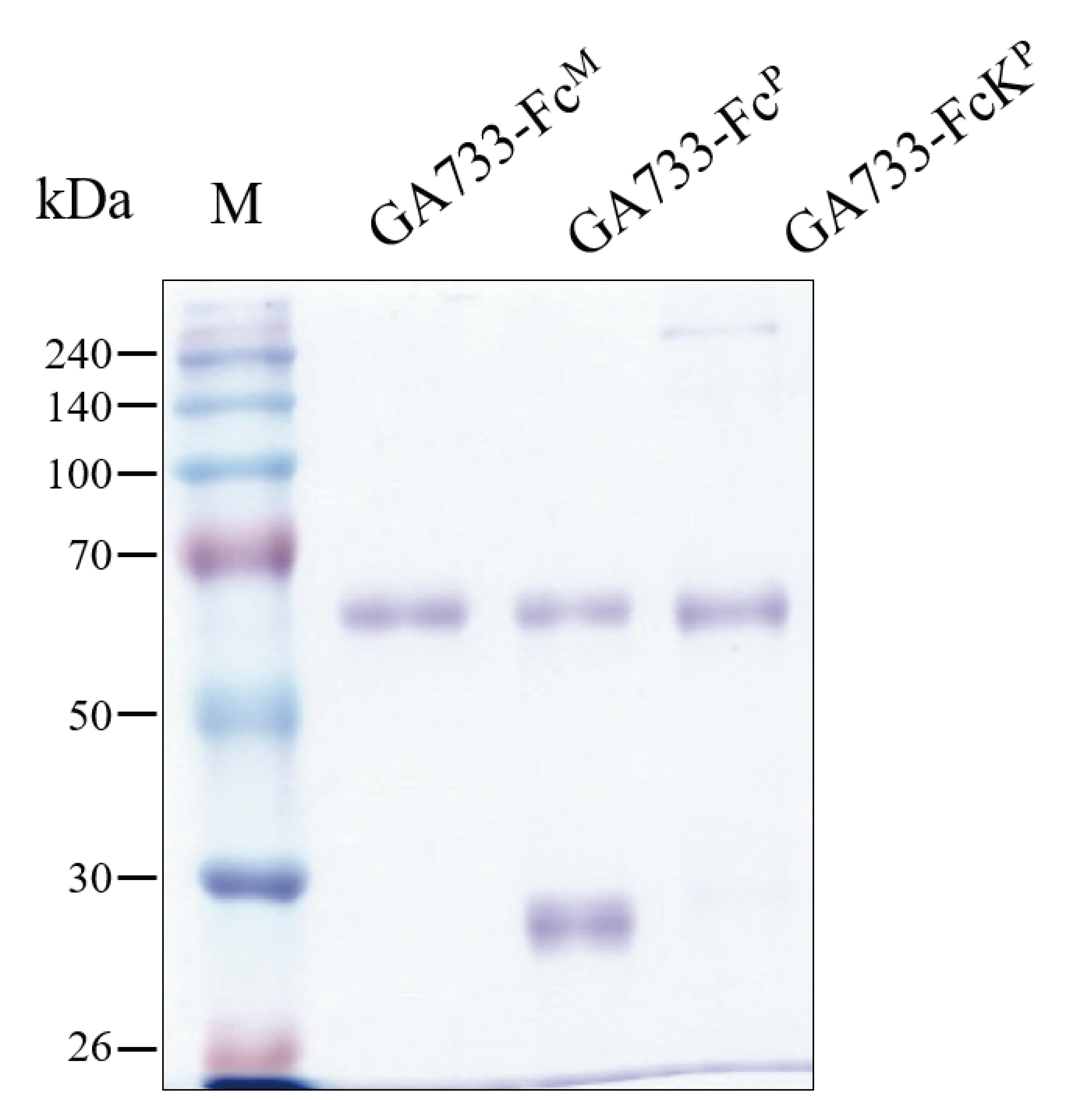

Supplement: Supplemental Information 16 — The Coomassie blue stained SDS-PAGE gel with GA733-Fc M, GA733-Fc P, and GA733-Fc P to confirm the equal amount for ELISA. This was used for Figure 5A. Loading of the same quantity of GA733-Fc P and GA733-FcK P proteins in ELISA was confirmed by SDS-PAGE. Lane 1, protein marker; Lane 2, positive control (+), mammalian-derived GA733-Fc (GA733-Fc M); Lane 3, GA733-Fc P; Lane 4, GA733-FcK P. [file peerj-09-10851-s016.png]
